# Overexpression of TMEM158 contributes to ovarian carcinogenesis

**DOI:** 10.1186/s13046-015-0193-y

**Published:** 2015-08-04

**Authors:** Zhongping Cheng, Jing Guo, Li Chen, Ning Luo, Weihong Yang, Xiaoyan Qu

**Affiliations:** Department of Obstetrics and Gynecology, Yangpu Hospital, Tongji University School of Medicine, Shanghai, 200090 China; Institute of Gynecological Minimally Invasive Medicine, Tongji University School of Medicine, Shanghai, 200090 China

**Keywords:** TMEM158, Ovarian cancer, Cell invasion, TGF-β, Cell adhesion

## Abstract

**Background:**

Transmembrane protein 158 (TMEM158) is a recently identified upregulated gene during Ras-induced senescence. Its association with various cancers has been recently reported. However, the expression and biological function of TMEM158 in ovarian cancer is still unclear. This study was aimed to elucidate the roles of TMEM158 in cell proliferation, adhesion and cell invasion of ovarian cancer cells.

**Methods:**

We analyzed TMEM158 mRNA level in ovarian cancer tissues and adjacent no-tumorous tissues by real-time PCR. We then suppressed TMEM158 expression of ovarian cancer cells by RNA interference and examined the effects of TMEM158 knockdown on cancerous transformation of ovarian cancer cells.

**Results:**

The RNA-sequencing data of the ovarian cancer cohort from The Cancer Genome Atlas project (TCGA) and our real-time PCR data showed that TMEM158 was overexpressed in ovarian cancer. Knockdown of TMEM158 by RNA interference in ovarian cancer cells significantly inhibited cell proliferation, which may be due to the increase of G1-phase arrest. Silencing of TMEM158 also inhibited cell adhesion, cell invasion as well as tumorigenicity in nude mice. Moreover, knockdown of TMEM158 notably repressed cell adhesion via down-regulating the expression intercellular adhesion molecule1 (ICAM1) and vascular cell adhesion molecule1 (VCAM1). Transforming Growth Factor-β (TGF-β) signaling pathway was also remarkably impaired by TMEM158 silencing.

**Conclusions:**

Our data suggests that TMEM158 may work as an oncogene for ovarian cancer and that inhibition of TMEM158 may be a therapeutic strategy for ovarian cancer.

## Introduction

Ovarian cancer is the most lethal type of gynecologic malignancy [1]. Due to its insidious onset, ovarian cancer is often diagnosed in advanced stages, resulting in a poor survival rate [2]. The five-year survival of patients with ovarian cancer is almost 30 % and has not significantly changed over the past 30 years regardless of important advances in surgery, radiation and chemotherapy [3–7]. This emphasizes the need for new diagnosis markers and therapies based on a well understanding of the molecular mechanisms underlying the oncogenesis and development of ovarian cancer.

Transmembrane protein 158 (TMEM158) was identified as an upregulated gene during Ras-induced senescence in human diploid fibroblasts infected with a RasV12-containing retrovirus and also known as Ras-induced senescence 1(RIS-1) [8]. In a non-small cell lung cancer (NSCLC) line transfected with the gene TLSC1, TMEM158 expression was found upregulated and tumor properties of the cells were suppressed [9]. There are a few studies concerned about the expression and functions of TMEM158 in tumors [10–12]. Gene expression analysis of ductal carcinoma showed that TMEM158 expression was not detected in tumor cells, but its expression was observed in the immediately adjacent stromal cells [11]. TMEM158 was overexpressed in Wilms tumors with somatic CTNNB1 mutations, suggesting a relationship between the Ras and Wnt signaling pathways [12]. TMEM158 has recently been identified as a target gene in the mutator pathway of microsatellite instability (MSI)-positive colorectal cancers. Low frequency MSI tumors with TMEM158 mutated usually were associated with a worse prognosis [10]. However, little is known about the expression pattern and biological functions of THEM158 in ovarian cancers.

To investigate the roles of TMEM158 in ovarian cancer, we determined its expression in ovarian cancer and normal tissues. The effects of TMEM158 knockdown in the proliferation, adhesion and invasion of ovarian cancer cells were assessed. The involved possible mechanism was also explored. Our study provides original documentation for the overexpression of TMEM158 in ovarian cancer and it may be an effective therapeutic target for this disease.

## Materials and methods

### Bioinformatics analysis

From The Cancer Genome Atlas (TCGA, https://tcga-data.nci.nih.gov/tcga/), we collect expression data of 568 ovarian cancers and 8 adjacent normal tissues. TMEM158 expression was shown to be statistically normally distributed by Shapiro-Wilk test. Levene’s test indicated heterogeneous variances of TMEM158 expression among ovarian cancers and normal tissues (*P* < 0.001).

To further investigate the biological pathways involved in ovarian cancer pathogenesis through TMEM158 pathway, we performed a gene set enrichment analysis (GSEA) [13, 14]. The KEGG gene sets biological process database (c2.KEGG.v4.0) were used for enrichment analysis.

### Cancer specimens

Tumor tissues and paired noncancerous tissues were collected from 25 patients diagnosed with Stage II/III epithelial ovarian serous adenocarcinoma, who were admitted to Department of Obstetrics and Gynecology, Yangpu Hospital, Tongji University (Shanghai, China) between 2010 and 2013. Written informed consent was obtained from all patients prior to participation on the study. The ethics committee of Tongji University (Shanghai, China) approved the design and informed consent.

### Cell lines

All culture media contained 10 % fetal bovine serum (FBS, Life Technologies), 100 mg/ml penicillin G, and 50 μg/ml streptomycin (Life Technologies). All cell lines were from American Type Culture Collection. The ovarian cancer cell lines OVCAR3, A2780 and HO-8910 cells were cultured in RPMI 1640 (Life Technologies). The CAOV3, SK-OV-3 and HEK 293T cells were cultured in DMEM (Life Technologies). All cells were maintained at 37 °C in 5 % CO_2_.

### RNA extraction and Real-time PCR

Total RNA was extracted using TRIzol Reagent (Invitrogen) according to the manufacturer’s instructions. Complementary DNA was synthesized with CDNA synthesis kit (Thermo Fisher Scientific). Real-time PCR was performed to detect mRNA levels of indicated genes. GAPDH was served as an internal control. The primers used were list as follows: TMEM158, 5′-TGTGCTTCGTGCTGTAGTTATC-3′ and 5′- TCAGTCCAAGGGCTTAAACATC-3′; GAPDH, 5′- AATCCCATCACCATCTTC -3′ and 5′-AGGCTGTTGTCATACTTC-3′; transforming Growth Factor-β (TGF-β), 5′- GACTACTACGCCAAGGAGGTC-3′ and 5′-GAGAGCAACACGGGTTCAG-3′; bone morphogenetic protein 4 (BMP4), 5′- CTGACCACCTCAACTCAAC -3′ and 5′-ACCCACATCCCTCTACTAC -3′; intercellular adhesion molecule1 (ICAM1), 5′-GTTGTTGGGCATAGAGAC-3′ and 5′- CAGGGCAGTTTGAATAGC-3′; vascular cell adhesion molecule1 (VCAM1), 5′-TGGGAACGAACACTCTTAC-3′ and 5′- CAGCAACTGAACACTTGAC - 3′. All reactions were conducted on an ABI 7300 real-time PCR machine (Applied Biosystems) using the following cycling parameters, 95 °C for 10 min, followed by 40 cycles of 95 °C for 15 s, 60 °C for 45 s. The gene expression was calculated using the ΔΔ Ct method. All data represent the average of three replicates.

### RNA interference and construction of stable cell line

Three shRNAs targeting position 1520 − 1540 (AAATGACCAAATCCTGTGTAT; named TMEM158-Ri-1), position 1627 − 1647 (TAAGAGAAGCTCTTTGTATCT; named TMEM158-Ri-2) and position 1719 − 1739 (TAACACCGATATATTGTTACC; named TMEM158-Ri-3) of human TMEM158 mRNA were cloned into a lentiviral vector (PLKO.1). A non-specific scramble shRNA sequence (CCTAAGGTTAAGTCGCCCTCG) was used as negative control. The constructs were then transfected into HEK293T cells with lentiviral packaging vectors by using lipofectamine 2000 (Invitrogen) according to the manufacture’s instruction. Viruses were collected 48 h after transfection and used to infect A2780 cells and HO-8910 cells. Stable cell line was generated by puromycin (Sigma) selection.

### Western blot

Total cell lysates were collected with radioimmunoprecipitation assay buffer (50 mmol/l Tris-HCl, 150 mmol/l NaCl, 1 % Triton X-100, 0.1 % SDS, 1 % deoxycholic acid sodium). Protein concentration was measured by BCA protein assay kit (Thermo Fisher Scientific). The supernatants with equal amounts of protein were separated on SDS-PAGE gels followed by electroblotting to nitrocellulose membranes. Western blot analysis was then carried out with appropriate primary and horseradish peroxidase-conjugated secondary antibodies. Membranes were developed with enhanced chemiluminescence (Bio-Rad). Antibodies against TMEM158, TGFβ1, BMP4, ICAM1 and VCAM1 were purchased from Abcam. GAPDH antibody was from CST Biotech. (Danvers, MA, USA). GAPDH was served as an internal control. The band intensity was measured using ImageJ software (National Institutes of Health).

### Cell proliferation assay

Cell proliferation was detected by using the Cell Count Kit-8 (CCK-8, Dojindo Laboratories). Briefly, indicated cells were plated onto 96-well plates (3 × 10^3^ cells per well). At indicated time point, CCK8 solution (10 μl in 100μl DMEM medium) was added to each well and incubated for 1 h. Optical density values (OD) was measured at wavelength 450 nm by a microplate reader (Bio-Rad). Each assay was carried out in triplicate.

### Cell cycle analysis

The cell cycle was evaluated by flow cytometry using propidium iodide (PI, Sigma, St. Louis, MO, USA) staining on a flow cytometer (BD Biosciences). Briefly, indicated cells were plated in 6-well plates. The cells were collected and fixed in 70 % ethanol at -20 °C overnight after 24 h culture. Cells were then washed in PBS and resuspended in staining solution containing 20 μg/ml PI and 200 μg/ml RNAse A. Experiments were performed in triplicate and 3 × 10^4^ cells were analyzed per sample. G1, S, and G2/M fractions were quantified using CellQuest software (BD Biosciences) and manual gating. Each assay was carried out in triplicate.

### Cell adhesion assay

The adhesion assay was performed in 12-well plates. The plates were pre-coated with 1 ml of fibronectin (5 μg/ml) for 2 h at room temperature. Cells were seeded into the coated plates at a density of 10^5^ cells per well and allowed to adhere at 37 °C for 1 h. Nonadherent cells were washed off with phosphate-buffered saline (PBS) and fixed in 4 % paraformaldehyde and stained with GIEMSA solution. The number of adherent cells determined as described previously [15]. Each assay was carried out in triplicate.

### *In vitro* invasion assay

The upper well of the transwell (Corning, NY, USA) was coated with Matrigel (BD Biosciences) at 37 °C in a 5 % CO2 incubator for 1 h. Indicated cells were serum starved for 24 h, and then 500 μl of cell suspension containing 10^5^ cells/ml were placed in the upper compartment of the chamber. Culture medium supplemented with 10 % FBS (750 μl) was added into the lower well of the chamber. The plates were incubated for 48 h. At the end of the incubation, the cells on the upper surface of the filter were completely removed by wiping with a cotton swab. Cells that migrated into the lower well were washed with PBS, fixed in 4 % paraformaldehyde and stained by 0.2 % crystal violet. Cells were photographed and counted under microscopy. Each assay was carried out in triplicate.

### *In vivo* tumorigenicity assay

Male BALB/c nude mice aged 4 − 5 weeks old were purchased from Shanghai Laboratory Animal Company. The mice were housed in a pathogen-free animal facility and randomly assigned to the control or experimental group (five mice per group). For each cell line, 2 × 10^6^ cells were resuspended in 200μl medium and subcutaneously injected into the nude mice. Tumor formation was monitored every three or four days by measuring the largest and the smallest diameter of the formed tumors, and the volume of the tumors was calculated using the following formula: volume = 1/2 × (largest diameter) × (smallest diameter)^2^. At euthanasia, the tumors were recovered and the wet weights of each tumor were examined. Animal care practices and all experiments were reviewed and approved by the Committee on the Ethics of Animal Experiments of Tongji University (Shanghai, China).

### Statistical analysis

The data were analyzed using the two-tailed Student’s *t*-test to calculate the statistical significance of difference between groups. The results were presented as the mean value ± SEM. Statistically significant differences were defined as having a *P* < 0.05.

## Results

### TMEM158 was overexpressed in ovarian cancer

**Fig. 1:**
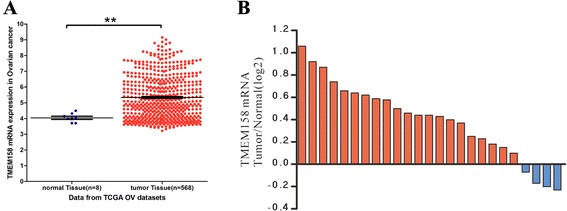
Overexpression of TMEM158 in ovarian cancer. (**a**) RNA-Seq analysis of TMEM158 mRNA expression in ovarian cancer and normal tissues. RNA-Seq analysis used data download from TCGA. ** *P* value < 0.01. (**b**) The mRNA level of TMEM158 in 25 pairs of ovarian tumor and normal tissue was detected by real-time PCR. Positive log2 (Tumor/Normal) on the y-axis indicated increased expression of TMEM158 in tumor tissue while negative log2 indicated decreased expression of TMEM158 in tumor tissue. TMEM158 mRNA was significantly overexpressed in ovarian tumor tissues as compared with normal tissues (*P* < 0.001)

To explore the expression of TMEM158 in ovarian cancer, we compared its expression by analyzing high throughput RNA-sequencing data of the ovarian cancer cohort from The Cancer Genome Atlas project (TCGA, https://tcga-data.nci.nih.gov/tcga/). As shown in Fig. 1a, TMEM158 expression was significantly increased in ovarian cancer tissues as compared with the adjacent tissues, which indicated that TMEM158 may be an oncogene in ovarian cancer.

To further determine TMEM158 expression in ovarian cancer, we performed real-time PCR analysis on 25 pairs of ovarian cancer and their matched noncancerous tissue samples. An overexpression of TMEM158 was found in 84 % (21/25) of tested ovarian cancer tissues (Fig. 1b). Statistical analysis using the student’s *t*-test showed that TMEM158 mRNA was significantly overexpressed in ovarian tumor tissues when compared with normal tissues (*P* < 0.001).

### TMEM158 was down-regulated by RNA interference (RNAi) in ovarian cancer cells

**Fig. 2:**
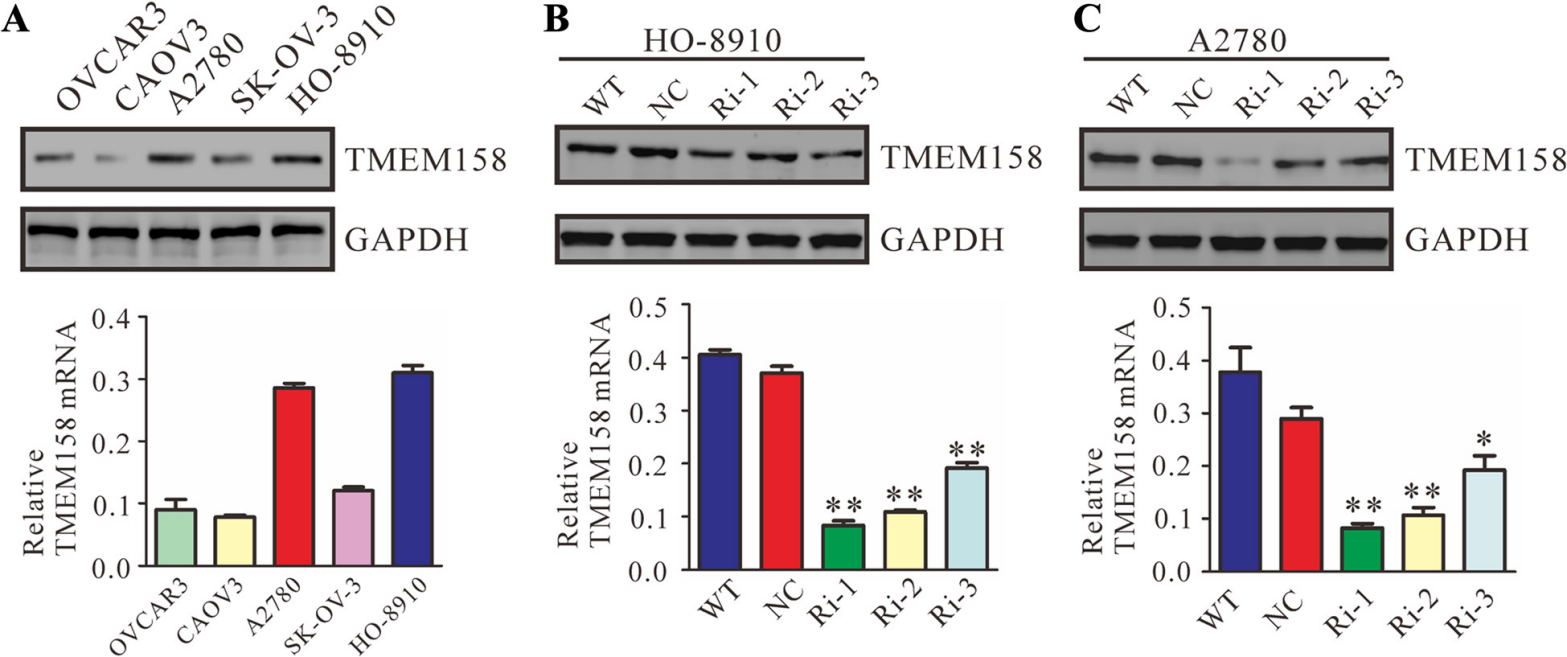
TMEM158 expression was suppressed by RNAi in ovarian cancer cells. (**a**) TMEM158 expression in five ovarian cancer cell lines was detected by Western blotting and real-time PCR. GAPDH was used as internal control. Highest expression of TMEM158 were observed in A2780 and HO-8910 cells, which were chosen for further analysis. Western blot (upper panel) and real-time PCR (lower panel) analysis showing the efficiency of TMEM158 knockdown in HO-8910 (**b**) and A2780 cells (**c**). WT: wild type cells; NC: scrambled shRNA virus infected cells; Ri-1, Ri-2 and Ri-3: TMEM158-shRNA-1, -2 and -3 virus infected cells

We then detected the protein and mRNA levels of TMEM158 in five ovarian cancer cell lines by Western blotting and real-time PCR, respectively. A high level of TMEM158 was observed in HO-8910 and A2780 cells (Fig. 2a). Therefore, these two cells were chosen for the following assays.

To investigate the functions of TMEM158 on ovarian cancer, shRNA plasmids were constructed for the suppressing of TMEM158 expression. Three pairs of human TMEM158 gene shRNA sequences and negative control (NC, a non-specific scramble shRNA sequence) were cloned into a lentiviral plasmid. The recombinant lentivirus was packaged in HEK293T cells. HO-8910 and A2780 cells were then infected with TMEM158-RNAi or NC virus. The silencing effect of TMEM158-RNAi were validated in HO-8910 (Fig. 2b) and A2780 cells (Fig. 2c) by Western-blotting and real-time PCR. Our results indicated that TMEM158-Ri-1 was the most efficient one and chosen for the further assays.

### Down-regulation of TMEM158 inhibited cell proliferation and induced G1-phase cell cycle arrest in ovarian cancer cells

**Fig. 3:**
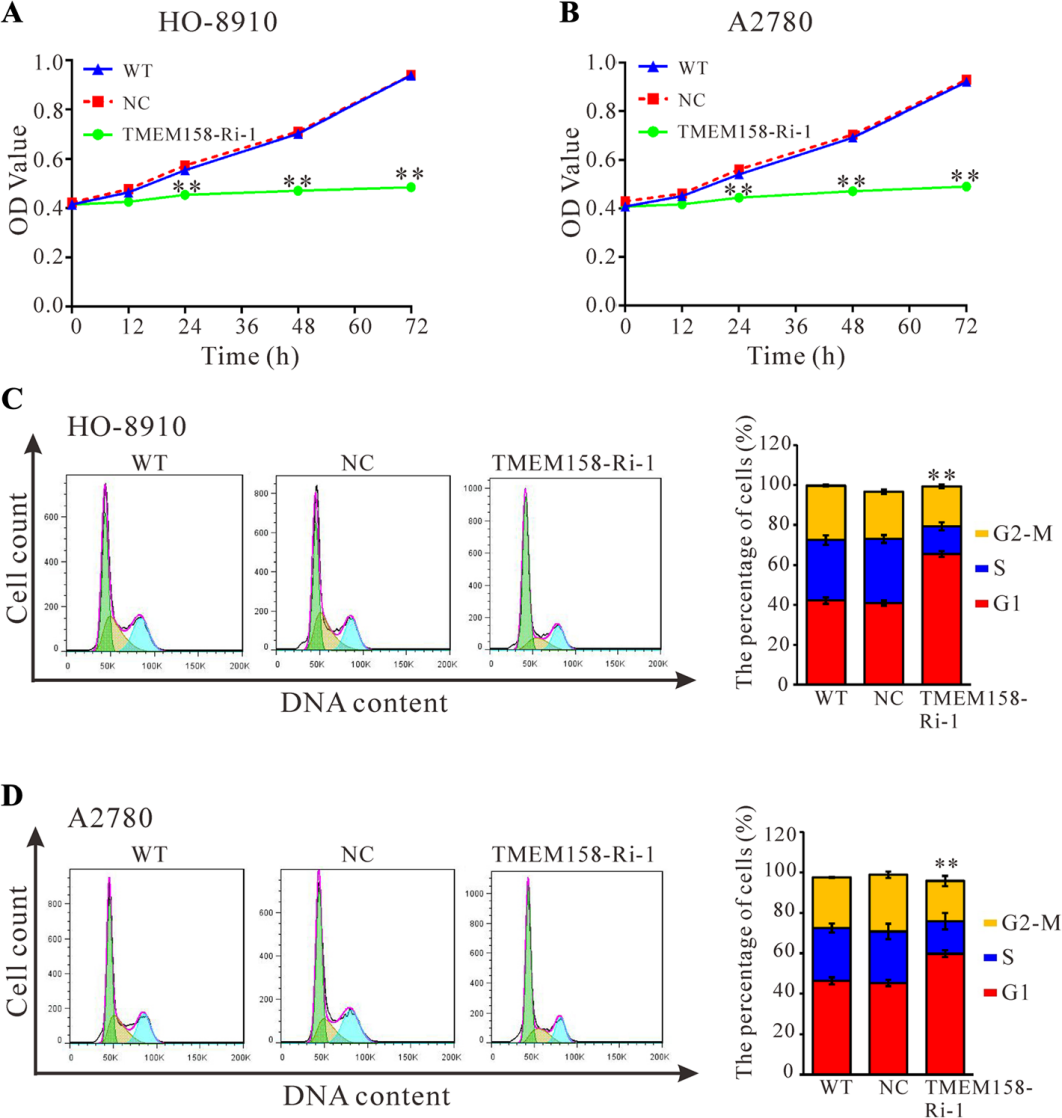
TMEM158 knockdown impaired cell proliferation and cell cycle in HO-8910 (**a** and **c**) and A2780 cells (**b** and **d**). Results of CCK-8 assay performed in control cells and TMEM158 knockdown cells were shown in (**a** and **b**). The percentage of cells in G1, S and G2-M phase for each sample at 48 h after viral infection were shown in (**c** and **d**). Data were based on at least 3 independent experiments, and shown as mean ± SEM. WT: wild type cells; NC: scrambled shRNA virus infected cells; TMEM158-Ri-1: TMEM158-shRNA-1 virus infected cells. Data were shown as the mean value ± SEM. **P < 0.01, as compared with NC cells

To substantiate the role of TMEM158 downregulation on cell proliferation, we detected the proliferation of ovarian cells infected with TMEM158-Ri-1 by using CCK-8 assay. As shown in Fig. 3a and b, cell growth was remarkably impaired in TMEM158-Ri-1 virus-infected cells (TMEM158-Ri-1) compared to wild-type cells (WT) and scramble shRNA virus-infected cells (NC). Our data suggested that TMEM158 was involved in the regulation of ovarian cancer cell proliferation.

The possible inhibitory effect of TMEM158 knockdown on cell cycle progression was then determined. As shown in Fig. 3c, downregulation of TMEM158 expression resulted in a higher number of HO-8910 cells in the G1 phase (65.4 % ± 1.5 %), compared with WT cells (42.1 % ± 1.6 %) and NC cells (40.9 % ± 1.2 %). Similar results were observed in A2780 cells (Fig. 3d). Our data suggested that TMEM158 knockdown notably induced G1-phase cell cycle arrest in ovarian cancer cells, which may lead to the inhibition of cell proliferation.

### TMEM158 knockdown inhibited cell adhesion

**Fig. 4:**
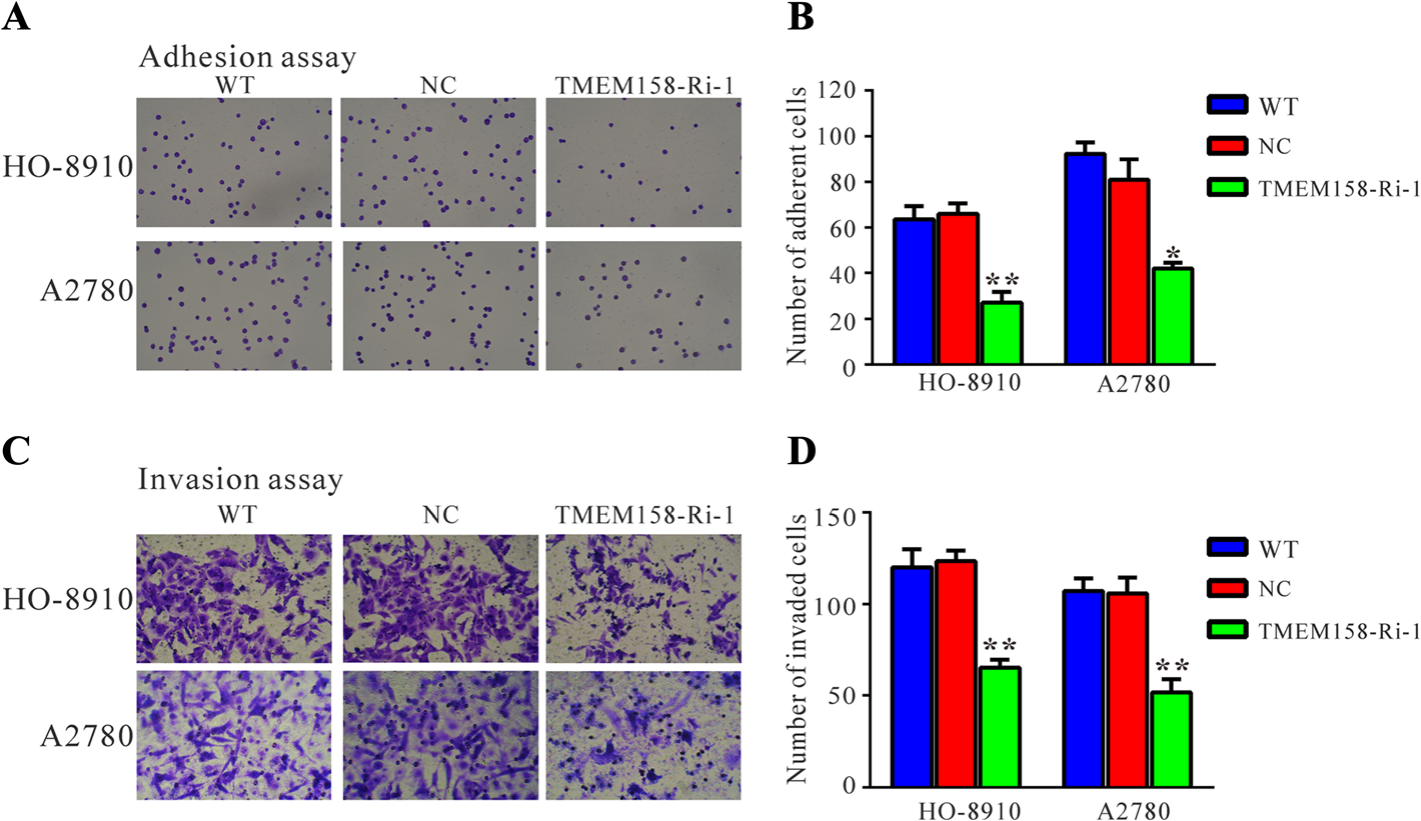
TMEM158 knockdown inhibited cell adhesion and cell invasion. We performed cell adhesion and invasion assay in fibronectin pre-coated plates and Matrigel-coated transwell chambers, respectively. Adherent cells or invaded cells were stained, photographed and counted. Representative images were shown in (**a**) and (**c**). Quantitative results of cell adhesion assay and invasion assay were shown in (**b**) and (**d**), respectively. Data were based on at least 3 independent experiments, and shown as mean ± SEM. WT: wild type cells; NC: scrambled shRNA virus infected cells; TMEM158-Ri-1: TMEM158-shRNA-1 virus infected cells. Data were shown as the mean value ± SEM. *P < 0.05, **P < 0.01 as compared with NC cells

The effects of TMEM158 on cell adhesion capacity were evaluated by cell adhesion assay (Fig. 4a and b). The adherent ability to fibronectin was significantly inhibited in ovarian cells by TMEM158 knockdown. The number of adherent TMEM158-Ri-1 cells was reduced to 35 % of that of NC cells when HO-8910 cells were used (Fig. 4a and b). Similar results were obtained in A2780 cells. These data suggested a role of TMEM158 in ovarian cancer cell adhesion.

### Suppressing TMEM158 expression inhibited the invasiveness of ovarian cancer cells

To explore whether TMEM158 affected the invasive ability of ovarian cancer cells, Matrigel-coated membranes chamber invasion assay was carried out. Dramatically reduced invasive ability was observed in TMEM158 knockdown cells compared to control cells. The number of invaded TMEM158-Ri-1 cells was decreased to 58 % of that of NC cells when HO-8910 cells were used (Fig. 4c and d). Similar results were observed in A2780 cells.

### TMEM158 knockdown inhibits ovarian cancer growth in nude mice xenograft model

**Fig. 5:**
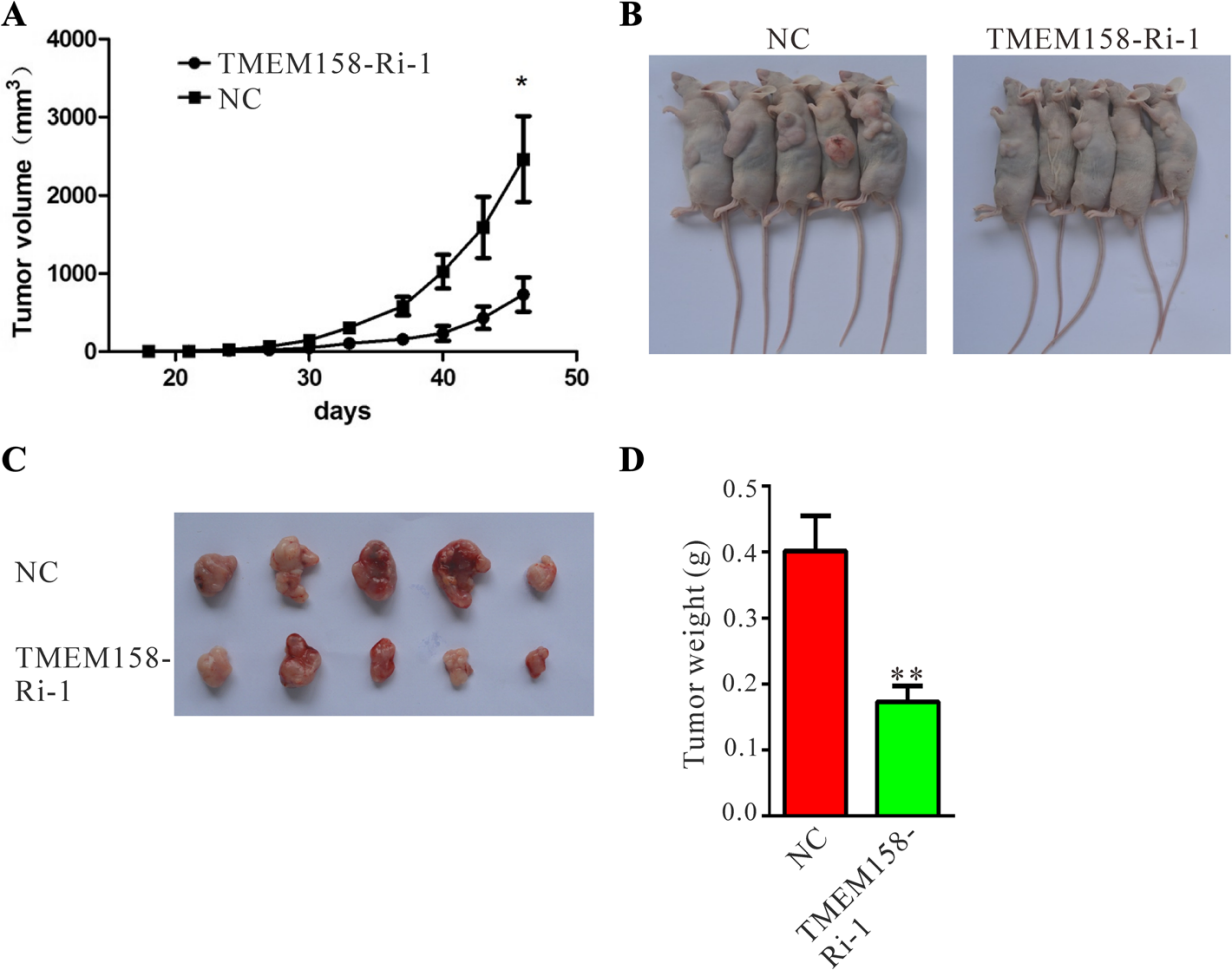
Knockdown of TMEM158 in HO-8910 cells inhibited tumorigenicity in nude mice. (**a**) An equal number of cells (NC and TMEM158-Ri-1) were inoculated subcutaneously into nude mice. Tumor volume was estimated based on the following equation: volume = 1/2 × (largest diameter) × (smallest diameter) ^2^. The mean value ± SEM. were from five animals in each group. (**b**) The nude mice with tumor formations. (**c**) Photograph of tumors derived from NC and TMEM158-Ri-1 cells in nude mice. (**d**) Weights of tumors. *P < 0.01 as compared with NC cells

Next, we determined whether knockdown of TMEM158 in ovarian cells could reduce tumor growth in vivo. HO-8910 cells stable transfected with scramble shRNA control or TMEM158-Ri-1 were subcutaneously injected in athymic nude mice as previously described [16], and tumor volumes were measured for 45 days. As shown in Fig. 5, the TMEM158-Ri-1 treated tumors grew slower than the control tumors in mice and the volume as well as weight was half that of control tumors. These data suggested that knockdown of TMEM158 inhibited tumor growth in nude mice.

### TMEM158 was positively correlated with TGF-β signaling pathway and cell adhesion molecules

**Fig. 6:**
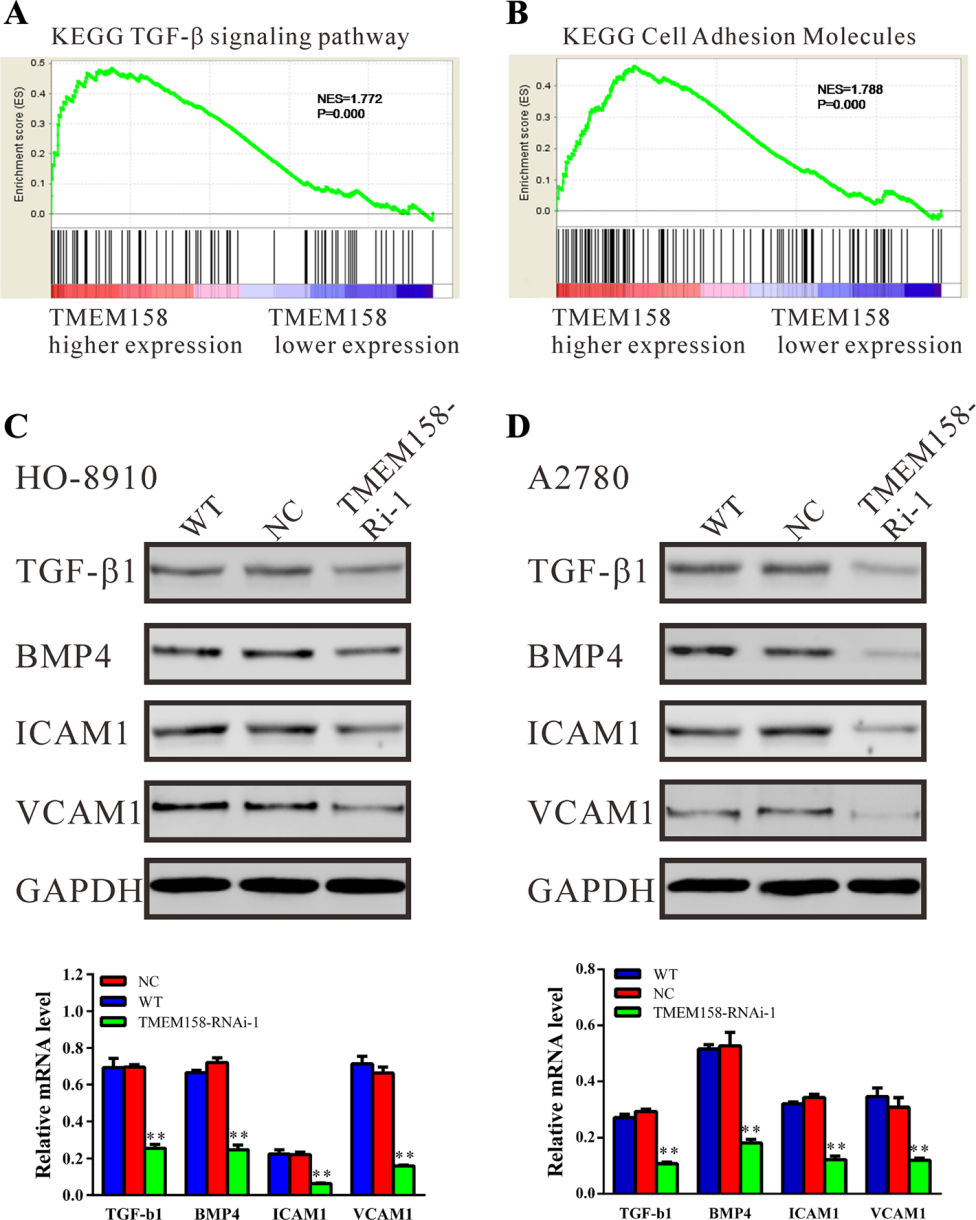
Effect of TMEM158 knockdown on the protein expressions of TGF-β1, BMP4, ICAM1 and VCAM1. (**a**, **b**) Identification of TGF-β signaling pathway and Cell adhesion genes as regulatory targets of TMEM158. Gene-set enrichment analysis (GSEA) identified significant association between TMEM158 and TGF-β signaling pathway or Cell adhesion genes in TCGA ovarian cancer dataset. Western blot (upper panel) and Real-time PCR (lower panel) analysis identified that expression of TGF-β1, BMP4, ICAM1 and VCAM1 was significantly decreased by TMEM158 RNAi in HO-8910 (**c**) and A2780 cells (**d**). Data were based on at least 3 independent experiments, and shown as mean ± SEM. **P < 0.01, as compared with NC cells

The exact pathway that TMEM158 may regulate in ovarian cancers remains unclear. To probe the TMEM158-associated pathways on an unbiased basis, we performed GSEA using high throughput RNA-sequencing data of the ovarian cancer cohort from TCGA. Among all the 188 predefined ‘KEGG pathways’ gene sets, TGF-β signaling pathway (Fig. 6a) and cell adhesion molecules (Fig. 6b) was identified to be closely correlated with TMEM158 expression in the TCGA ovarian cancer dataset. Gene expression of important regulators in TGF-β signaling pathway and cell adhesion molecules was determined both in mRNA and protein levels (Fig. 6c and d). The expression of TGF-β, BMP4, ICAM1 and VCAM1 was remarkably decreased after downregulation of TMEM158.

## Discussion

Several investigation have been reported regarding the expression and functions of TMEM158 in different tumors [9–12]. In the present study, analysis using high throughput RNA-sequencing data from TCGA demonstrated a higher expression of TMEM158 in ovarian cancer compared to normal tissues (Fig. 1a). We then found that TMEM158 mRNA expression was elevated in 84 % (21/25) of tested ovarian cancer tissues by real-time PCR (Fig. 1b). To further investigate the functions of TMEM158 in ovarian cancer, we suppressed the expression of TMEM158 in two ovarian cells, HO-8910 and A2780 cells by RNA interference (Fig. 2b and c). Our data showed that suppressing of TMEM158 expression notably inhibited the proliferation, cell cycle progression (Fig. 3), adhesion, invasion (Fig. 4) and tumorigenicity of ovarian cells (Fig. 5). Taken together, our data further indicated contribution of TMEM158 in ovarian cell carcinogenesis.

The regulation of cell cycle is frequently abnormal in most common malignancies, resulting in aberrant cell proliferation [17, 18]. Here, silencing of TMEM158 in ovarian cancer cells significantly promoted cell arrest in G1-phase (Fig. 3c and d), which may lead to the inhibition of cell proliferation (Fig. 3a and b). Our results were consistent with the previous study that found that TMEM158 was an upregulated gene in Ras-senescent human fibroblasts and expected to negatively regulate cell cycle [8]. However, another study indicated that TMEM158-deficient mouse embryonic fibroblasts showed no apparent change in cellular functions, such as proliferation, senescence and oncogenic transformation [19]. The discrepancy may due to differences in cell types and experimental models.

The exact pathway that TMEM158 may regulate in ovarian cancers remains unclear. Our GSEA data indicated that TMEM158 overexpression was positively correlated with the TGF-β signaling pathway (Fig. 6a). As we all known, TGF-β signaling participates in a diverse set of cellular processes, including cell proliferation, differentiation, apoptosis, and specification of developmental fate [20]. The TGF-β signaling pathway has also been considered as a promoter of tumor progression and invasion. In response to elevated TGF-β levels, the tumor cell becomes more migratory and invasive [21, 22]. BMP4 is up-regulated in human colonic adenocarcinoma and human colorectal cancer. HCT116 overexpressing BMP4 exhibited enhanced migration and invasion characteristic [23]. In ovarian cancer, TGF-β and BMP4 are reported to play an important role in controlling ovarian cancer metastasis [24]. In the presents study, TMEM158 knockdown significantly decreased the expression of TGF-β and BMP4 (Fig. 6c and d), which indicated a relation between TMEM158 function and the regulation of TGF-β signaling in ovarian cancer cells.

Moreover, our GSEA data indicated that TMEM158 overexpression was positively correlated with cell adhesion molecules (Fig. 6b). It is reported that serum levels of two important adhesion molecules, ICAM1 and VCAM1 are significant higher in patients with colorectal cancer, ovarian cancer, NSCLC and breast cancer [25–28]. ICAM1 and VCAM1 are considered to be important in the process of malignant tumor growth [26, 29]. In the presents study, TMEM158 knockdown remarkably decreased the mRNA and protein levels of ICAM1 and VCAM1. The dowregulation of ICAM1 and VCAM1 may be associated with the impaired cell-matrix adhesion and invasive ability (Fig. 4) of TMEM158 RNAi cells. To our knowledge, our data firstly associated the functions of TMEM158 with cell adhesion molecules.

Taken together, we found the overexpression of TMEM158 in ovarian cancer cells, which was associated with cancerous transformation. Our study firstly associates TMEM158 with the regulation of cell adhesion molecules and TGF-β signaling pathway, thus may provide useful information for targeted therapy. Whether TMEM158 can be used as a potential therapeutic target for ovarian cancer remains to be further investigated.
